# Elevated Mean Corpuscular Hemoglobin Concentration as a Potential Peripheral Biomarker of Parkinson’s Disease: A Pilot Case–Control Study in a Mexican Population

**DOI:** 10.3390/brainsci15090966

**Published:** 2025-09-06

**Authors:** Ernesto Gerardo Miranda-Morales, Elizabeth Romero-Gutierrez, Francisco Xavier Castellanos-Juárez, Edna Madai Méndez-Hernández, Alma Cristina Salas-Leal, Osmel La Llave-León, Gerardo Quiñones-Canales, Ada Sandoval-Carrillo, José Manuel Salas-Pacheco, Oscar Arias-Carrión

**Affiliations:** 1 Instituto de Investigación Científica, Universidad Juárez del Estado de Durango, Durango 34000, Mexico; ernesto.miranda@mail.com (E.G.M.-M.); xavier_castellanos@hotmail.com (F.X.C.-J.); edna_madai@hotmail.com (E.M.M.-H.); alma.salas@ujed.mx (A.C.S.-L.); olallavel@ujed.mx (O.L.L.-L.); adda-sandoval@hotmail.com (A.S.-C.); 2 División de Neurosciencias, Clínica, Instituto Nacional de Rehabilitación Luis Guillermo Ibarra Ibarra, Mexico City 14389, Mexico; romguteliz@gmail.com; 3 Hospital Santiago Ramón y Cajal, Instituto de Seguridad y Servicios Sociales de los Trabajadores del Estado (ISSSTE), Durango 34070, Mexico; gqc55@hotmail.com; 4 Tecnologico de Monterrey, Escuela de Medicina y Ciencias de la Salud, Mexico City 14380, Mexico

**Keywords:** Parkinson’s disease, red blood cells, mean corpuscular hemoglobin concentration, pilot case–control study, peripheral biomarkers, neuroinflammation

## Abstract

**Background**: Alterations in peripheral red blood cell (RBC) indices have been proposed as potential biomarkers for Parkinson’s disease (PD), but their diagnostic utility and relation to clinical features remain uncertain. **Methods**: We conducted a pilot case–control study involving 70 PD patients and 122 controls from two neurology centers in Mexico. Standardized hematology analyses provided RBC indices, and neuropsychiatric assessments included the Hamilton Depression Rating Scale (HAM-D) and Mini-Mental State Examination (MMSE). Associations between RBC indices and PD were tested using multivariable logistic regression adjusted for age, sex, and smoking. Diagnostic performance was evaluated by receiver operating characteristic (ROC) curve analysis. Subgroup analyses stratified PD patients by age at onset, disease duration, and Hoehn and Yahr (HY) stage. **Results**: PD patients exhibited significantly higher mean corpuscular hemoglobin (MCH) and mean corpuscular hemoglobin concentration (MCHC) than controls. Elevated MCHC was independently associated with PD (OR = 1.68, 95% CI 1.35–2.09; *p* < 0.001). Sex-stratified models confirmed consistent associations in women (OR = 1.57) and men (OR = 1.79). ROC analysis demonstrated fair diagnostic accuracy for MCHC (AUC 0.72, 95% CI 0.65–0.80; cutoff 33.9 g/dL, sensitivity 62.9%, specificity 72.1%). Sex-specific thresholds improved sensitivity in women (90.6%) and specificity in men (74.6%). Within the PD group, MCHC did not differ by HY stage or disease duration, and showed no correlation with UPDRS, HAM-D, or MMSE scores. Early-onset cases (<50 years) showed numerically higher MCHC, though numbers were limited. **Conclusions**: This pilot study confirms that an elevated MCHC is independently associated with PD, a finding consistent across both sexes and independent of disease severity. MCHC demonstrates fair diagnostic performance, supporting its potential as a low-cost, accessible biomarker. Larger longitudinal studies integrating RBC indices with inflammatory and iron-regulatory markers are warranted to establish their role in the diagnosis and differential diagnosis of PD. Elevated MCHC was associated with PD, and an MCHC-based index (cutoff 33.9 g/dL; AUC 0.72, sensitivity 62.9%, specificity 72.1%) showed potential as a simple diagnostic marker.

## 1. Introduction

Parkinson’s disease (PD) is a multisystem neurodegenerative disorder affecting over 10 million people worldwide, with hallmark motor symptoms—bradykinesia, rigidity, resting tremor—and a diverse range of non-motor manifestations, including depression, cognitive decline, and autonomic dysfunction [1,2,3,4]. While the cardinal pathological features include dopaminergic neuronal loss in the substantia nigra and alpha-synuclein aggregation [5], evidence suggests that PD is not confined to the central nervous system but also involves systemic alterations, including blood, immune, and metabolic dysregulation [6,7].

Inflammation has emerged as a central component of PD pathogenesis, interacting with mitochondrial dysfunction, oxidative stress, and iron dyshomeostasis [8]. Peripheral immune and inflammatory changes—including altered cytokine signatures—have also been reported in atypical parkinsonian disorders such as progressive supranuclear palsy (PSP), where distinct interleukin patterns contribute to differential diagnosis [9]. Against this backdrop, red blood cell (RBC) indices may provide accessible markers that reflect both iron regulation and low-grade systemic inflammation, complementing molecular biomarkers in the stratification of PD from other forms of parkinsonism [10].

Despite advances in neuroimaging, genetics, and biomarker discovery, the diagnosis of PD remains fundamentally clinical, often delayed until motor features emerge, by which point significant neurodegeneration has already occurred [10,11,12]. Reliable, low-cost peripheral biomarkers that support early detection, track disease progression, or inform stratified care are urgently needed. In this context, non-specific inflammatory parameters such as the neutrophil-to-lymphocyte ratio (NLR) and related indices have recently been highlighted in parkinsonisms, underscoring the potential added value of combining RBC indices with broader immune and iron panels in future work [10].

Peripheral hematological parameters, particularly those related to erythropoiesis and iron metabolism, have garnered increasing interest in PD research. An association between anemia and subsequent PD risk has been identified in large cohort studies, with reduced hemoglobin and iron deficiency preceding clinical diagnosis by years or even decades [13,14,15]. Transcriptomic and proteomic analyses of blood samples from PD patients further reveal dysregulation in genes linked to hemoglobin biosynthesis, erythroid differentiation, and iron transport, suggesting a systemic signature of disrupted iron homeostasis [16,17,18].

Beyond their passive diagnostic value, red blood cells and hemoglobin may play active roles in PD pathogenesis. Hemoglobin has been detected within dopaminergic neurons, localized to mitochondria, and implicated in modulating oxidative phosphorylation and redox balance [19]. Red blood cells also carry the majority of circulating alpha-synuclein, raising the possibility of peripheral involvement in the handling and propagation of this pathogenic protein [20,21]. Nevertheless, the clinical relevance of RBC indices in PD remains poorly defined, especially in relation to disease subtypes and sex differences. Emerging evidence suggests that PD motor subtypes (tremor-dominant, postural instability/gait difficulty, and indeterminate) may exhibit distinct biological profiles. At the same time, sex-specific factors, such as estrogen’s role in iron metabolism and differing trajectories of nigrostriatal degeneration, could shape peripheral readouts [22].

Although red cell parameters such as mean corpuscular volume (MCV), mean corpuscular hemoglobin (MCH), and mean corpuscular hemoglobin concentration (MCHC) are widely available in routine clinical practice, their utility as PD biomarkers has received little systematic evaluation. Prior studies report conflicting results, often without adjusting for relevant confounding factors or assessing diagnostic accuracy [23]. To date, most have focused on anemia and iron deficiency, while the potential diagnostic performance of elevated RBC indices (e.g., via ROC analysis) remains underexplored [24].

In this pilot case–control study, we examined RBC indices in individuals with PD and matched controls, with particular focus on MCH and MCHC. We assessed their independent association with PD, tested for sex-specific and clinical subgroup effects, and evaluated their diagnostic performance using ROC analysis. We also explored correlations with motor severity, depressive symptoms, and cognitive performance. Our findings provide new insights into peripheral hematological changes in PD, highlighting the potential of RBC indices as accessible, non-invasive biomarkers to support diagnosis, differential diagnostic work-up, and future precision medicine approaches. In particular, we propose an MCHC-based index (cutoff 33.9 g/dL; AUC 0.72) that may facilitate simple risk stratification in clinical practice.

## 2. Methods

### 2.1. Study Design and Participant Recruitment

We conducted a pilot case–control study involving 70 individuals with Parkinson’s disease (PD) and 122 control participants, consecutively recruited between 2022 and 2024 from the neurology departments of General Hospital 450 and General Hospital Santiago Ramón y Cajal–ISSSTE in Durango, Mexico. Eligibility for the PD group required a clinical diagnosis of PD according to the UK Parkinson’s Disease Society Brain Bank (UKPDSBB) diagnostic criteria. Control participants were drawn from the same hospital population and were confirmed to be free of neurodegenerative and major psychiatric disorders.

All participants were enrolled during routine outpatient visits. Written informed consent was obtained prior to participation in the study. Each participant underwent a structured interview to collect demographic and lifestyle data. Cognitive function was evaluated with the Mini-Mental State Examination (MMSE), and depressive symptoms were assessed using the Hamilton Depression Rating Scale (HAM-D). For PD patients, disease severity was quantified using the Unified Parkinson’s Disease Rating Scale (UPDRS) and staged according to the Hoehn and Yahr (HY) scale.

### 2.2. Ethical Considerations

The study protocol was reviewed and approved by the ethics committees of both participating institutions: ISSSTE Hospital General Dr. Santiago Ramón y Cajal (approval number: EeI/056/13) and Hospital General 450, Durango (approval number: 23/08/12). All procedures were conducted in accordance with national research regulations and the ethical principles outlined in the Declaration of Helsinki (1975, revised 2000).

### 2.3. Blood Sampling and Laboratory Analysis

Fasting venous blood samples were collected using the BD Vacutainer^®^ system (REF 368861; K2 EDTA 7.2 mg, Becton Dickinson, Franklin Lakes, NJ, USA). Approximately 4 mL of whole blood was collected and processed within 24 h. Analyses were performed with a Hemat 18 Licon automated hematology analyzer (Licon, Tlalnepantla, Mexico), which requires 10 μL of sample per run. The analyzer was operated under standardized quality control procedures, and internal performance was monitored using Levey–Jennings control plots.

The analyzer generated complete blood counts, including red blood cell indices (hemoglobin, hematocrit, red blood cell count, mean corpuscular volume [MCV], mean corpuscular hemoglobin [MCH], and mean corpuscular hemoglobin concentration [MCHC]), as well as white blood cell and platelet counts. Sex-specific comparisons were performed through stratified analyses, and separate correlation matrices were constructed for men and women.

### 2.4. Statistical Analysis

All statistical analyses were performed using IBM SPSS Statistics for Windows, Version 21.0 (IBM Corp., Armonk, NY, USA), and complemented with R (version 4.3) for advanced modeling and diagnostic analyses. Continuous variables were tested for normality using the Kolmogorov–Smirnov test and compared using the Student’s *t*-test or the Mann–Whitney U test, as appropriate. Categorical data were analyzed using the Chi-square test. Statistical significance was defined as *p* < 0.05.

Pearson’s correlation coefficients were calculated to assess associations between hematological parameters, depression (HAM-D scores), cognition (MMSE scores), and clinical severity (HY and UPDRS scores).

To evaluate the association between red blood cell indices and PD, multivariable logistic regression models were constructed. Model 1 included red cell indices (MCH and MCHC); Model 2 additionally adjusted for age, sex, and smoking; and Model 3 was a fully adjusted model incorporating all covariates. To explore potential sex-related heterogeneity, an interaction term (sex × MCHC) was added, and sex-stratified logistic regression models were performed. Odds ratios (OR) with 95% confidence intervals (CI) were reported.

To assess the diagnostic performance of MCHC, receiver operating characteristic (ROC) curve analysis was performed. The area under the curve (AUC) was estimated with a 95% CI obtained by bootstrap resampling (2000 iterations). The optimal cutoff was identified using Youden’s *J* statistic, and corresponding sensitivity and specificity were reported. ROC curves were also constructed separately for men and women.

Within the PD group, subgroup analyses were conducted to examine whether MCHC differed according to clinical characteristics. Patients were stratified by:

- age at onset (<50 vs. ≥50 years),
- disease duration (≤5 vs. >5 years), and
- HY stage (early 1–2 vs. mid-to-late 3–5).

Group comparisons were performed using Welch’s *t*-test, and effect sizes were expressed as Hedges’ *g*. Correlations of MCHC with disease duration and HY stage were additionally tested with Pearson’s *r*.

## 3. Results

### 3.1. Participant Characteristics

A total of 192 individuals were included: 70 patients with Parkinson’s disease (PD) and 122 controls. The two groups were comparable in age (70.1 ± 10.4 vs. 69.5 ± 8.8 years; *p* = 0.651) and sex distribution (54.3% vs. 51.6% male; *p* = 0.839). PD patients had significantly higher HAM-D scores (10.9 ± 7.3 vs. 8.5 ± 5.8; *p* = 0.022), while MMSE scores were similar between groups (25.3 ± 5.0 vs. 25.6 ± 3.6; *p* = 0.703). Within the PD group, the mean age at onset was 64.7 ± 10.7 years, disease duration was 5.4 ± 4.4 years, UPDRS score was 73.7 ± 34.1, and Hoehn and Yahr (HY) stage was 2.7 ± 1.1 (Table 1).

### 3.2. Red Blood Cell Indices in PD and Control Groups

Compared with controls, PD patients exhibited significantly higher mean corpuscular hemoglobin (MCH) (31.1 ± 1.9 vs. 30.5 ± 1.7 pg; *p* = 0.012) and mean corpuscular hemoglobin concentration (MCHC) (34.1 ± 1.6 vs. 32.8 ± 1.7 g/dL; *p* < 0.001). Sex-stratified analyses confirmed elevated MCHC in both women (33.8 vs. 32.6 g/dL; *p* = 0.001) and men (34.3 vs. 33.0 g/dL; *p* < 0.001). MCH was significantly higher in male PD patients (31.8 vs. 30.6 pg; *p* = 0.001), but not in females (Table 2 and Supplementary Table S2; Table 3 and Supplementary Table S3).

### 3.3. Multivariable Models and Sex Interaction

In unadjusted logistic regression, higher MCHC was associated with increased odds of PD (OR = 1.66 per 1 g/dL increase; 95% CI 1.34–2.06; *p* < 0.001). This association remained robust after adjustment for age, sex, and smoking (OR = 1.68; 95% CI 1.35–2.09; *p* < 0.001) (Table 4, Supplementary Table S1). Addition of a sex × MCHC interaction term showed no significant effect modification (OR = 1.13; 95% CI 0.73–1.75; *p* = 0.583). However, sex-stratified models confirmed consistent associations: OR = 1.57 (95% CI 1.17–2.12; *p* = 0.003) in women and OR = 1.79 (95% CI 1.29–2.47; *p* < 0.001) in men.

### 3.4. Diagnostic Performance of MCHC

**Figure 1:**
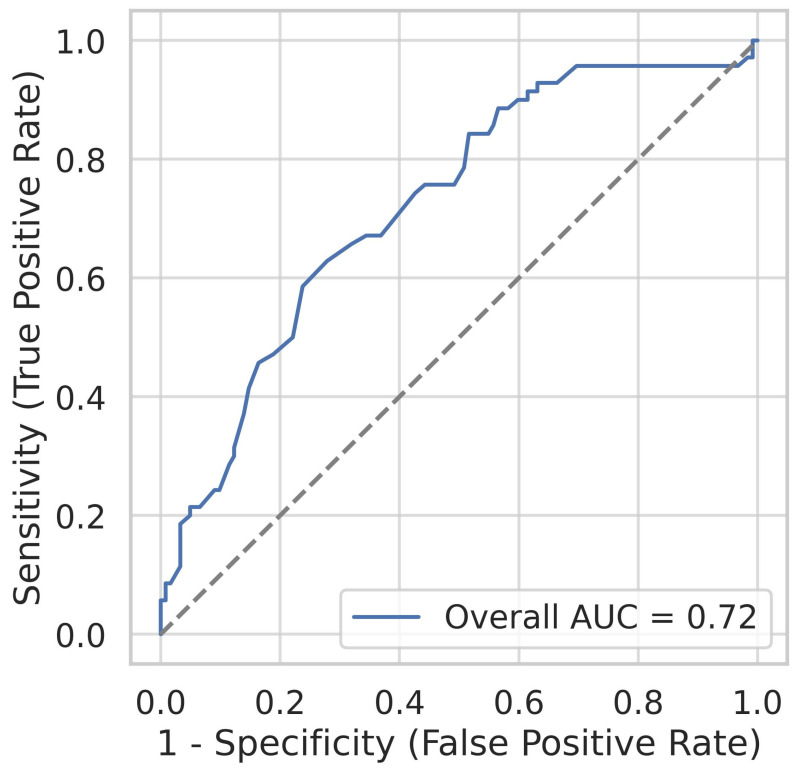
Receiver Operating Characteristic (ROC) Curve of MCHC for Discrimination Between PD Cases and Controls. The ROC curve demonstrates the diagnostic performance of mean corpuscular hemoglobin concentration (MCHC) in distinguishing patients with Parkinson’s disease (PD) from healthy controls. The area under the curve (AUC) was 0.72 (95% CI, 0.65–0.80), with an optimal cutoff of 33.9 g/dL corresponding to a sensitivity of 62.9% and a specificity of 72.1%.

ROC analysis demonstrated that MCHC discriminated PD from controls with an AUC of 0.72 (95% CI 0.65–0.80). The optimal cutoff of 33.9 g/dL yielded a sensitivity of 62.9% and specificity of 72.1% (Table 5, Supplementary Table S6). In sex-specific analyses, the AUC was 0.71 in women (cutoff 32.5 g/dL; sensitivity 90.6%; specificity 49.2%) and 0.75 in men (cutoff 34.0 g/dL; sensitivity 68.4%; specificity 74.6%) (Figure 1).

### 3.5. Stratified Analyses Within PD

**Figure 2:**
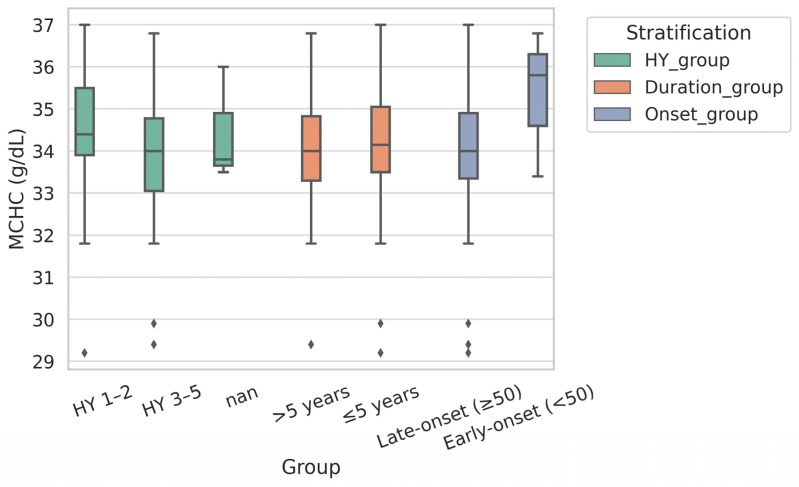
Stratified MCHC Levels Among PD Patients. Boxplots show MCHC values stratified by clinical subgroups within the PD cohort: (A) early-stage (HY 1–2) versus mid-to-late-stage disease (HY 3–5); (B) shorter (≤5 years) versus longer (>5 years) disease duration; and (C) early-onset (<50 years) versus late-onset (≥50 years) PD. MCHC levels did not differ significantly across HY stage or disease duration subgroups, and were numerically higher in the early-onset PD group; however, this group was underpowered (*n* = 3). Boxes represent interquartile ranges, horizontal lines indicate medians, whiskers denote the 1.5× interquartile range, and outliers are plotted individually. nan (Not a Number) indicates that statistical comparison or effect size estimation could not be computed due to the very small sample size in the early-onset subgroup (*n* = 3).

MCHC was further examined in relation to clinical features of PD (Table 5, Supplementary Table S6, Figure 2).

HY stage: MCHC did not differ significantly between early-stage (HY 1–2: 34.3 ± 1.6 g/dL) and mid-to-late-stage PD (HY 3–5: 34.0 ± 1.6 g/dL; *p* = 0.375). Correlation analysis confirmed no linear association (r = −0.03; *p* = 0.799).

Disease duration: Patients with disease duration of ≤5 years and >5 years had similar MCHC values (34.2 vs. 34.0 g/dL; *p* = 0.768), with no correlation to duration (r = −0.03; *p* = 0.828).

Age at onset: Early-onset (<50 years) cases showed numerically higher MCHC (35.3 ± 1.7 g/dL) compared to late-onset (≥50 years) (34.1 ± 1.6 g/dL), although this difference did not reach significance (*p* = 0.331) due to the small size of the early-onset subgroup (*n* = 3).

### 3.6. Correlations with Clinical Scores

No significant correlations were observed between MCHC and UPDRS, HAM-D, or MMSE scores, suggesting independence from motor severity, depression, and cognition (Supplementary Tables S4 and S5). Hemoglobin and hematocrit, however, were inversely correlated with depressive symptoms in female and male subgroups.

## 4. Discussion

In this pilot case–control study, we demonstrate that elevated MCHC is independently associated with PD in a Mexican population. After adjusting for age, sex, and smoking, MCHC remained a significant predictor of PD, with consistent effect sizes across both sexes. ROC analysis showed that MCHC achieved fair diagnostic performance (AUC 0.72; sensitivity 62.9%; specificity 72.1%), with slightly different optimal thresholds in women (32.5 g/dL) and men (34.0 g/dL). Notably, MCHC values did not differ significantly across HY stages or by disease duration, supporting the hypothesis that this parameter is independent of clinical severity. Together, these findings suggest that MCHC may serve as a peripheral, readily available biomarker with potential value in the early identification of PD.

### 4.1. Pathophysiological Context: Inflammation and Iron Biology

Our results should be interpreted within the broader context of PD pathophysiology, which extends beyond nigrostriatal degeneration to encompass systemic inflammatory and metabolic changes [22,23,25]. Neuroinflammation, mitochondrial dysfunction, and oxidative stress closely interact with iron homeostasis, providing a plausible biological link to alterations in erythropoiesis and RBC indices. Elevated MCHC may reflect subtle shifts in iron handling and redox balance, as hemoglobin concentration within red cells is influenced by systemic iron metabolism and inflammatory signals. Notably, inflammatory profiles have also been described in atypical parkinsonian syndromes such as progressive supranuclear palsy–parkinsonism predominant (PSP-P), where interleukin patterns distinguish subtypes [9]. This raises the possibility that RBC indices, when combined with immune markers, could contribute to differential diagnosis across parkinsonian disorders.

### 4.2. Sex Differences and Biological Interpretation

Although sex × MCHC interaction analyses did not reveal significant effect modification, sex-stratified models confirmed consistent associations in both women (OR 1.57) and men (OR 1.79). These findings align with established biological differences: estrogen regulates iron metabolism [22,23] and may shape erythrocyte indices in women, while men exhibit faster dopaminergic degeneration and distinct trajectories of PD progression [25]. Our data suggest that while MCHC is a robust correlate across sexes, sex-specific cutoffs may enhance diagnostic performance, consistent with other biomarker fields where thresholds differ by biological sex.

### 4.3. Clinical Subgroups and Diagnostic Value

Stratified analyses within the PD cohort revealed that MCHC was not associated with HY stage or disease duration, thereby reinforcing its independence from motor severity and supporting its potential as a trait marker rather than a state marker. Although early-onset PD cases (<50 years) exhibited numerically higher MCHC, the subgroup was too small (*n* = 3) to draw firm conclusions. Future studies with larger and stratified samples are needed to clarify whether age at onset or motor subtype influences RBC indices. Given that PD motor subtypes (tremor-dominant, PIGD, indeterminate) may represent biologically distinct entities [3], it will be important to explore whether MCHC varies across subtypes or interacts with non-motor features such as depression and cognition. In our cohort, MCHC showed no correlation with UPDRS, HAM-D, or MMSE scores, indicating that it may provide information independent of motor and neuropsychiatric symptom burden.

### 4.4. Comparison with Inflammatory Indices

Recent work has highlighted the neutrophil-to-lymphocyte ratio (NLR) and related indices as accessible markers of systemic inflammation in PD and other parkinsonisms [10]. Although our dataset did not include differential leukocyte counts, which precluded NLR analysis, this represents an important avenue for future research. Integrating MCHC with broader immune–inflammatory markers (NLR, platelet-to-lymphocyte ratio, systemic immune–inflammation index) and iron-regulatory measures (ferritin, transferrin saturation, hepcidin, soluble transferrin receptor) could provide a multimarker panel that improves diagnostic accuracy and differential diagnosis.

### 4.5. Strengths and Limitations

The strengths of this study include its rigorous design, case–control structure, adjustment for confounders (age, sex, smoking), sex-stratified analyses, and multiple subgroup evaluations (onset age, HY stage, disease duration). To our knowledge, this is the first study to demonstrate an independent association between elevated MCHC and PD in a Mexican population, supported by diagnostic ROC analyses.

Nonetheless, several limitations must be acknowledged. First, the sample size is modest, and early-onset PD cases were underrepresented, necessitating replication in larger cohorts. Second, iron metabolism markers and differential leukocyte counts were not collected, which prevented the assessment of inflammatory and iron-regulatory indices. Third, medication histories were not available, limiting our ability to assess whether dopaminergic or other therapies influence RBC indices [26]. Finally, the cross-sectional design prevents conclusions about temporality—whether MCHC changes precede PD onset or reflect secondary processes remains unknown. For these reasons, we explicitly classify this work as a pilot case–control study.

### 4.6. Potential Index and Conceptual Model

The findings of this pilot case–control study support the role of MCHC as a candidate diagnostic index for PD. Multivariable logistic regression confirmed that an elevated MCHC was independently associated with PD (OR = 1.68 per 1 g/dL increase; 95% CI, 1.35–2.09; *p* < 0.001), a finding consistent across both sexes. ROC curve analysis yielded an AUC of 0.72 (95% CI, 0.65–0.80), indicating fair discriminatory performance. The optimal threshold of 33.9 g/dL provided a sensitivity of 62.9% and specificity of 72.1%. Notably, sex-stratified analyses improved diagnostic performance, with a cutoff of 32.5 g/dL in women yielding high sensitivity (90.6%), albeit lower specificity (49.2%), while a cutoff of 34.0 g/dL in men achieved balanced accuracy (sensitivity 68.4%, specificity 74.6%). These values suggest that an MCHC-based index may contribute to risk stratification in clinical practice, particularly when used in conjunction with sex-specific thresholds.

To formalize its diagnostic use, two approaches can be considered. First, a probability-based model derived from logistic regression:

$$\begin{array}{c} logit\left(P\right)=\beta_{0}+0.52\times MCHC\\ P\left(PD\right)=\frac{1}{1+e^{-\left(\beta_{0}+0.52\times MCHC\right)}} \end{array}$$

where 0.52 = ln(1.68), corresponding to the odds ratio per 1 g/dL increase in MCHC. The intercept (*β*_0_) can be estimated in larger cohorts to allow the model to output individual-level PD probabilities.

Second, a simplified threshold-based index can be directly applied:

$$MCHC-Index=\begin{array}{l} 1\;if\;MCHC\geq 33.9\;g/dL\;(overall)\\ 1\;if\;MCHC\geq 32.5\;g/dL\;(women)\\ 1\;if\;MCHC\geq 34.0\;g/dL\;(men)\\ 0\;otherwise \end{array}$$

This provides a practical binary tool for risk stratification in clinical settings.

Beyond its diagnostic potential, elevated MCHC may also serve as a biological marker reflecting systemic mechanisms relevant to PD pathogenesis. The conceptual model presented here situates MCHC at the intersection of peripheral iron metabolism dysregulation and low-grade systemic inflammation. Both processes are closely linked to oxidative stress and mitochondrial dysfunction, which in turn contribute to nigrostriatal neurodegeneration. This framework supports the hypothesis that peripheral hematological indices are not merely bystanders but may capture early systemic alterations associated with PD. The integration of MCHC into multimarker panels, together with iron-regulatory measures (ferritin, transferrin saturation, hepcidin) and inflammatory indices (e.g., neutrophil-to-lymphocyte ratio, platelet-to-lymphocyte ratio, systemic immune–inflammation index), could substantially enhance diagnostic accuracy and aid in differential diagnosis across parkinsonian disorders.

### 4.7. Future Directions

Future studies should pursue longitudinal designs that integrate hematologic indices, iron-regulatory markers, and inflammatory parameters. Inclusion of validated PD subtyping and atypical parkinsonian cohorts (e.g., PSP-P) will clarify specificity and differential diagnostic value. Finally, larger multicenter samples are needed to refine sex-specific thresholds and validate MCHC as part of an accessible, low-cost biomarker panel for PD.

## 5. Conclusions

This pilot case–control study demonstrates that elevated mean corpuscular hemoglobin concentration (MCHC) is independently associated with Parkinson’s disease in a Mexican population. After adjustment for age, sex, and smoking, higher MCHC remained a significant correlate of PD, consistent across both sexes. Receiver operating characteristic (ROC) analysis confirmed fair diagnostic performance (AUC 0.72; optimal cutoff 33.9 g/dL, sensitivity 62.9%, specificity 72.1%). Sex-stratified thresholds improved diagnostic accuracy, with enhanced sensitivity in women and balanced specificity in men. Significantly, MCHC did not vary by Hoehn and Yahr stage, disease duration, or neuropsychiatric scores, supporting its role as a trait marker independent of clinical severity.

Based on these findings, we propose an MCHC-based index to support risk stratification in clinical practice:

Overall threshold: 33.9 g/dL (AUC 0.72; sensitivity 62.9%; specificity 72.1%).

Women: 32.5 g/dL (sensitivity 90.6%; specificity 49.2%).

Men: 34.0 g/dL (sensitivity 68.4%; specificity 74.6%).

This accessible and low-cost index provides a first step toward incorporating red blood cell parameters into the diagnostic work-up of PD. While replication in larger, longitudinal, and multiethnic cohorts is essential, the present work highlights the potential of MCHC as a simple, non-invasive biomarker that reflects systemic processes relevant to PD pathogenesis. Integrating MCHC with iron-regulatory and inflammatory markers may ultimately yield a multimarker panel with improved diagnostic precision and differential diagnostic value.

## Figures and Tables

**Table 1:** Clinical and demographic characteristics of the study population.

Characteristic	Controls (*n* = 122)	PD Cases (*n* = 70)	*p*-Value
Age (years)	69.48 ± 8.80	70.14 ± 10.36	0.651
Male sex, *n* (%)	63 (51.6%)	38 (54.3%)	0.839
HAM-D score	8.45 ± 5.81	10.85 ± 7.27	0.022
MMSE score	25.56 ± 3.62	25.30 ± 4.96	0.703
Age at PD onset (years)	—	64.74 ± 10.67	—
Disease duration (years)	—	5.39 ± 4.39	—
UPDRS total score	—	73.7 ± 34.1	—
Hoehn & Yahr stage	—	2.7 ± 1.05	—

**Table 2:** Red Blood Cell Indices in Female PD Cases and Controls.

Parameter	Controls (*n* = 59)	PD Cases (*n* = 32)	*p*-Value
Hemoglobin (g/dL)	14.01 ± 1.24	14.11 ± 0.97	0.707
Hematocrit (%)	43.01 ± 3.79	41.88 ± 3.15	0.155
RBC count (×10^6^/µL)	4.64 ± 0.38	4.69 ± 0.41	0.587
MCV (fL)	92.63 (88.9–94.2)	90.00 (86.7–92.0)	0.014
MCH (pg/cell)	30.24 ± 1.88	30.37 ± 1.98	0.763
MCHC (g/dL)	32.64 (30.9–33.8)	33.84 (33.1–34.6)	0.001
WBC (×10^3^/µL)	6.04 ± 1.62	6.46 ± 1.35	0.216
Platelets (×10^3^/µL)	209.1 ± 56.7	227.0 ± 80.7	0.272

Values are mean ± SD or median (IQR).

**Table 3:** Red Blood Cell Indices in Male PD Cases and Controls.

Parameter	Controls (*n* = 63)	PD Cases (*n* = 38)	*p*-Value
Hemoglobin (g/dL)	15.27 ± 2.09	15.52 ± 1.69	0.416
Hematocrit (%)	46.36 ± 6.62	45.09 ± 4.40	0.153
RBC count (×10^6^/µL)	5.10 ± 0.69	4.87 ± 0.50	0.304
MCV (fL)	92.22 ± 8.78	92.37 ± 5.37	0.269
MCH (pg/cell)	30.63 (29.7–31.7)	31.75 (30.5–32.8)	0.001
MCHC (g/dL)	32.97 (31.6–34.1)	34.33 (33.5–35.7)	<0.001
WBC (×10^3^/µL)	6.82 ± 1.75	6.40 ± 1.64	0.236
Platelets (×10^3^/µL)	202.2 ± 63.4	202.4 ± 63.6	0.990

Values are mean ± SD or median (IQR).

**Table 4:** Multivariable Logistic Regression for Association Between MCHC and PD.

Model	Term	OR (95% CI)	*p*-Value
Model 1 (Unadjusted)	MCHC	1.66 (1.34–2.06)	<0.001
Model 2 (Adjusted)	MCHC	1.68 (1.35–2.09)	<0.001
	Age	1.02 (0.98–1.05)	0.358
	Sex	0.94 (0.48–1.83)	0.861
	Smoking	0.70 (0.32–1.50)	0.355
Model 3 (Interaction model)	MCHC	1.58 (1.17–2.13)	0.003
	Sex	0.02 (0.0–39,506)	0.578
	MCHC × Sex	1.13 (0.73–1.75)	0.583
	Age	1.02 (0.98–1.05)	0.338
	Smoking	0.70 (0.33–1.52)	0.373

OR = odds ratio per 1 g/dL increase in MCHC.

**Table 5:** Diagnostic Performance of MCHC and Stratified Analyses Within PD.

(**A**) **ROC Curve Analysis for MCHC**						
**Group**	**AUC (95% CI)**	**Optimal Cutoff (g/dL)**	**Sensitivity**	**Specificity**		
Overall	0.72 (0.65–0.80)	33.9	62.9%	72.1%		
Female	0.71	32.5	90.6%	49.2%		
Male	0.75	34.0	68.4%	74.6%		
(**B**) **Stratified Analyses Within PD Patients**						
**Stratification**	**Group 1**	**Mean MCHC (g/dL) ± SD**	**Group 2**	**Mean MCHC (g/dL) ± SD**	***p*-Value**	**Effect Size (Hedges’ g)**
HY stage	HY 1–2 (*n* = 25)	34.32 ± 1.62	HY 3–5 (*n* = 42)	33.96 ± 1.57	0.375	0.23
Disease duration	≤5 years (*n* = 42)	34.15 ± 1.62	>5 years (*n* = 28)	34.04 ± 1.53	0.768	0.07
Age at onset	Early < 50 y (*n* = 3)	35.33 ± 1.75	Late ≥ 50 y (*n* = 67)	34.05 ± 1.56	0.331	0.81 *

* Interpret with caution due to very small early-onset subgroup (*n* = 3).

## Data Availability

The data supporting the reported results are openly available in GitHub at: https://github.com/ExpNeuro/IIC-UJED-Hemoglobin-PDMX (accessed on 7 August 2025).

## Supplementary Materials

The following supporting information can be downloaded at: https://www.mdpi.com/article/10.3390/brainsci15090966/s1, Table S1: Logistic Regression Models for Association Between RBC Indices and PD; Table S2: Red Blood Cell Indices in Female PD Cases and Controls. Table S3: Red Blood Cell Indices in Male PD Cases and Controls. Table S4: Pearson Correlation Between Blood Parameters, Depression (HAM-D), and Cognition (MMSE) in Females. Table S5: Pearson Correlation Between Blood Parameters, Depression (HAM-D), and Cognition (MMSE) in Males. Table S6: Diagnostic Performance of MCHC and Stratified Analyses Within PD.

## Abbreviations

The following abbreviations are used in this manuscript:

AUC	Area under the curve
CI	Confidence interval
HAM-D	Hamilton Depression Rating Scale
Hct	Hematocrit
Hgb	Hemoglobin
HY	Hoehn and Yahr staging
MCV	Mean corpuscular volume
MCH	Mean corpuscular hemoglobin
MCHC	Mean corpuscular hemoglobin concentration
MMSE	Mini-Mental State Examination
NLR	Neutrophil-to-lymphocyte ratio
OR	Odds ratio
PD	Parkinson’s disease
PLR	Platelet-to-lymphocyte ratio
PSP-P	Progressive supranuclear palsy–Parkinsonism predominant
RBC	Red blood cell(s)
ROC	Receiver operating characteristic
SII	Systemic immune–inflammation index
UPDRS	Unified Parkinson’s Disease Rating Scale
WBC	White blood cell(s)
