# Supplementary material for: Elevated Mean Corpuscular Hemoglobin Concentration as a Potential Peripheral Biomarker of Parkinson’s Disease: A Pilot Case–Control Study in a Mexican Population
Source: Brain Sci. 2025 Sep 6;15(9):966. doi: 10.3390/brainsci15090966 (PMC12468377; doi:10.3390/brainsci15090966)
Supplement: Supplementary file 1 [file brainsci-15-00966-s001.zip › brainsci-3836169-supplementary.pdf]

Supplementary Table S1. Logistic Regression Models for Association Between RBC Indices and PD.

Parameter	Model 1 (Unadjusted) OR (95% CI), p	Model 2 (Adjusted for age, sex, smoking) OR (95% CI), p	Model 3 (Fully adjusted) OR (95% CI), p
**MCH**	1.04 (0.84–1.27), p = 0.70	—	1.03 (0.84–1.27), p = 0.72
**MCHC**	1.69 (1.29–2.05), p < 0.001	—	1.69 (1.30–2.08), p < 0.001
**Sex**	—	0.84 (0.45–1.56), p = 0.59	1.08 (0.55–2.12), p = 0.81
**Age**	—	1.00 (0.97–1.04), p = 0.60	1.01 (0.98–1.05), p = 0.37
**Smoking**	—	0.68 (0.33–1.39), p = 0.29	0.69 (0.32–1.50), p = 0.29

Footnote: Models were constructed incrementally. Model 1 includes red cell indices, Model 2 includes demographic covariates, and Model 3 includes both.

Supplementary Table S2. Red Blood Cell Indices in Female PD Cases and Controls.

Parameter	Controls (n = 59)	PD Cases (n = 32)	p-value
**Hemoglobin (g/dL)**	14.01 ± 1.24	14.11 ± 0.97	0.707
**Hematocrit (%)**	43.01 ± 3.79	41.88 ± 3.15	0.155
**RBC count ($\times 10^6$/μL)**	4.64 ± 0.38	4.69 ± 0.41	0.587
**MCV (fL)**	92.63 (88.9–94.2)	90.00 (86.7–92.0)	0.014
**MCH (pg/cell)**	30.24 ± 1.88	30.37 ± 1.98	0.763
**MCHC (g/dL)**	32.64 (30.9–33.8)	33.84 (33.1–34.6)	0.001
**WBC ($\times 10^3$/μL)**	6.04 ± 1.62	6.46 ± 1.35	0.216
**Platelets ($\times 10^3$/μL)**	209.1 ± 56.7	227.0 ± 80.7	0.272

Values are mean ± SD or median (IQR). p-values: [1] Student's t-test, [2] Mann–Whitney U test.

Supplementary Table S3. Red Blood Cell Indices in Male PD Cases and Controls.

Parameter	Controls (n = 63)	PD Cases (n = 38)	p-value
**Hemoglobin (g/dL)**	15.27 ± 2.09	15.52 ± 1.69	0.416
**Hematocrit (%)**	46.36 ± 6.62	45.09 ± 4.40	0.153
**RBC count (×10^6/μL)**	5.10 ± 0.69	4.87 ± 0.50	0.304
**MCV (fL)**	92.22 ± 8.78	92.37 ± 5.37	0.269
**MCH (pg/cell)**	30.63 (29.7–31.7)	31.75 (30.5–32.8)	0.001
**MCHC (g/dL)**	32.97 (31.6–34.1)	34.33 (33.5–35.7)	<0.001
**WBC (×10^3/μL)**	6.82 ± 1.75	6.40 ± 1.64	0.236
**Platelets (×10^3/μL)**	202.2 ± 63.4	202.4 ± 63.6	0.990

Values are mean ± SD or median (IQR). p-values: [1] Student's t-test, [2] Mann–Whitney U test.

Supplementary Table S4. Pearson Correlation Between Blood Parameters, Depression (HAM-D), and Cognition (MMSE) in Females.

	Hgb	Hct	MCV	MCH	MCHC	RBC	HAM-D	MMSE
**Hemoglobin**	1							
**Hematocrit**	0.789	1						
**MCV**	0.094	0.386	1					
**MCH**	0.349	0.081	0.602	1				
**MCHC**	0.308	-0.319	-0.446	0.409	1			
**RBC count**	0.695	0.677	-0.371	-0.366	0.030	1		
**HAM-D score**	-0.109	-0.134	0.005	0.007	-0.004	-0.133	1	
**MMSE score**	0.017	0.012	-0.058	-0.042	-0.007	0.079	-0.138	1

Values are Pearson's correlation coefficients (r). Boldface (not shown here) indicates statistical significance at p < 0.05.

Supplementary Table S5. Pearson Correlation Between Blood Parameters, Depression (HAM-D), and Cognition (MMSE) in Males.

	Hgb	Hct	MCV	MCH	MCHC	RBC	HAM-D	MMSE
**Hemoglobin**	1							
**Hematocrit**	0.915	1						
**MCV**	0.461	0.603	1					
**MCH**	0.300	0.122	0.407	1				
**MCHC**	0.136	-0.260	-0.431	0.394	1			
**RBC count**	0.222	0.222	-0.534	-0.167	0.069	1		
**HAM-D score**	-0.202	-0.196	-0.103	-0.103	0.043	-0.126	1	
**MMSE score**	0.190	0.142	-0.127	-0.016	0.126	0.212	-0.230	1

Values are Pearson's correlation coefficients (r). Boldface (not shown here) indicates statistical significance at $p < 0.05$.

Supplementary Table S6. Diagnostic Performance of MCHC and Stratified Analyses Within PD.

(A) ROC Curve Analysis for MCHC.

Group	AUC (95% CI)	Optimal cutoff (g/dL)	Sensitivity	Specificity
**Overall**	0.72 (0.65–0.80)	33.9	62.9%	72.1%
**Female**	0.71	32.5	90.6%	49.2%
**Male**	0.75	34.0	68.4%	74.6%

(B) Stratified Analyses of MCHC Within PD.

Stratification	Group 1	Mean MCHC ± SD (g/dL)	Group 2	Mean MCHC ± SD (g/dL)	p-value	Effect size (Hedges' g)
**HY stage**	HY 1–2 (n = 25)	34.32 ± 1.62	HY 3–5 (n = 42)	33.96 ± 1.57	0.375	0.23
**Disease duration**	≤5 years (n = 42)	34.15 ± 1.62	>5 years (n = 28)	34.04 ± 1.53	0.768	0.07
**Age at onset**	Early <50y (n = 3)	35.33 ± 1.75	Late ≥50y (n = 67)	34.05 ± 1.56	0.331	0.81*

*Interpret with caution due to the very small early-onset subgroup (n = 3); *nan* values in *Figure 2* indicate that statistical comparisons or effect size estimation could not be computed.
